# Polytrauma With an Open Three-Part Proximal Humeral Fracture and Ipsilateral Comminuted Olecranon Fracture in an Intoxicated Patient: Challenges in Management

**DOI:** 10.7759/cureus.111104

**Published:** 2026-06-18

**Authors:** Sarah Mahmood, Fares Kellany, Khadija Bano, Dana Ibrahim, Hassan Alrifai, Anas Obaid

**Affiliations:** 1 Medicine, Hull York Medical School, York, GBR; 2 Medicine, Medical University of Bahrain, Muharraq, BHR; 3 Medicine, University College of Medicine and Dentistry, Lahore, PAK; 4 Orthopedics, Saudi German Hospital, Dubai, ARE

**Keywords:** case report, dual plating, intoxicated patient, multimodal fixation, olecranon fracture, open fracture, orthopaedic trauma, polytrauma, proximal humeral fracture, trauma surgery

## Abstract

We report the case of a young male polytrauma patient who sustained an open three-part proximal humeral fracture with an associated ipsilateral comminuted olecranon fracture following a fall from height. On presentation, he was intoxicated and hemodynamically unstable, requiring immediate management according to Advanced Trauma Life Support principles.

Initial management included prompt resuscitation followed by urgent irrigation and debridement of the open fracture. Definitive fixation was performed in a staged manner after physiological optimization. The proximal humeral fracture was managed with dual plating and calcar support to restore stability and alignment, while the comminuted olecranon fracture required a multimodal fixation approach incorporating plates, cerclage wiring, and screw fixation to achieve stable reconstruction.

Despite the severity of injury and initial physiological compromise, the patient achieved a favorable outcome with preserved neurovascular function and early return of joint mobility. At the six-month follow-up, there was satisfactory functional recovery of both the shoulder and elbow. This case is notable for the rare combination of injuries and highlights the importance of systematic trauma management, careful operative planning, and a multidisciplinary approach to rehabilitation. It also underscores the ethical considerations involved in urgent decision-making when treating an intoxicated patient who lacks capacity.

## Introduction

Proximal humeral fractures account for approximately 5% to 6% of all fractures and are most commonly encountered in elderly, osteoporotic patients following low-energy mechanisms [1]. In younger individuals, these injuries typically result from high-energy trauma and are frequently accompanied by additional injuries [2]. Open fractures of the proximal humerus are distinctly uncommon but carry substantial challenges, including heightened risks of infection, avascular necrosis, and long-term functional compromise [3].

Simultaneous ipsilateral fractures of the proximal humerus and ulna are rarely described in the literature and present unique demands for surgical planning and fixation strategy. Management becomes further complicated in circumstances where the patient’s capacity for informed consent is impaired, such as intoxication, which requires careful navigation of ethical and legal considerations.

This case describes a polytrauma injury involving an open three-part proximal humeral fracture (Neer’s classification) [4] in association with a comminuted proximal ulna fracture. It illustrates the operative and multidisciplinary approach undertaken, highlighting both the technical aspects of fixation and the broader clinical challenges, with successful functional recovery achieved.

## Case presentation

A young male patient presented in October 2024 following a fall from a second-floor height (approximately 20 to 30 feet). He was managed as a polytrauma case. On arrival, the patient was intoxicated, conscious, but disoriented.

The primary survey per the Advanced Trauma Life Support (ATLS) principles showed a clear airway, normal breathing (peripheral oxygen saturation (SpO₂) 100% on room air), hypotension (BP 70/30 mmHg) with tachycardia (heart rate (HR) 130 bpm), indicating class III hemorrhagic shock, a Glasgow coma scale (GCS) score of 14, and equal and reactive pupils. The cervical spine was stabilized, with no thoraco-abdominal injuries. The secondary survey revealed an open right upper limb injury, classified as a Gustilo type 2 open fracture [5], with gross elbow deformity and a self-reported pain score of 8/10, intact distal pulses, and no neurological deficits. The patient required assistance with basic self-care. The systemic review excluded pneumothorax or hemothorax. Initial portable X-ray revealed a right proximal humeral fracture with an ipsilateral olecranon fracture (Figure 1). The CT scans confirmed a three-part proximal humeral fracture (Figure 2) with a comminuted olecranon fracture and a non-displaced fracture of the left sacrum (Figure 3).

**Figure 1:**
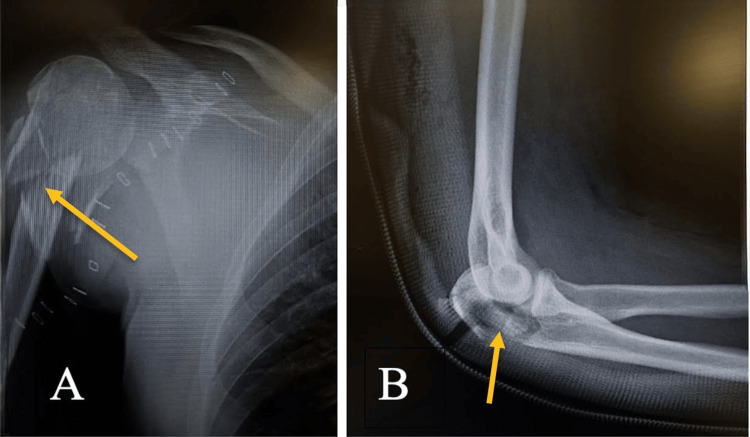
Radiograph of the right proximal humerus and olecranon fractures A: Anteroposterior shoulder displaying a right proximal humerus fracture, B: Lateral elbow displaying a right olecranon fracture

**Figure 2:**
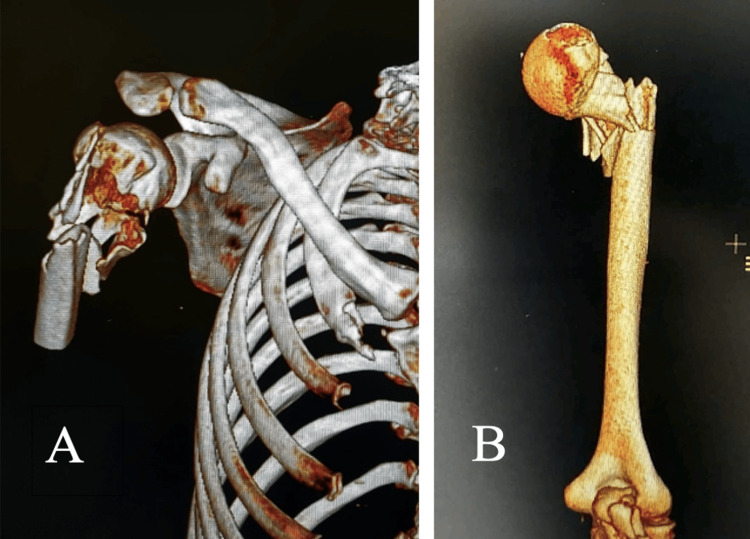
3D CT reconstructions demonstrating proximal humeral fracture pattern A: Shoulder girdle and thoracic cage showing a comminuted proximal humeral fracture, B: Humerus demonstrating a comminuted three-part fracture of the proximal humerus

**Figure 3:**
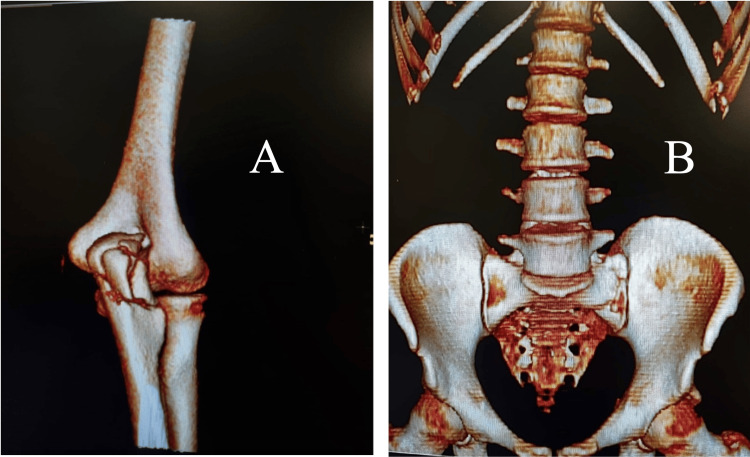
CT imaging demonstrating associated injuries A: Comminuted right olecranon fracture, B: Non-displaced fracture of the left sacrum

Given the severity of the injury, the patient underwent urgent irrigation and debridement within 24 hours of admission. As the patient was intoxicated and temporarily lacked decision-making capacity, his personal consent was deemed invalid. In line with institutional protocol, informed consent was therefore obtained from his sibling after a full explanation of the clinical situation, and further endorsed by two consultant surgeons prior to proceeding with surgery.

Definitive fixation was done approximately 48 hours after the initial debridement, following stabilization of the patient's condition and preoperational optimization. The surgery lasted five hours, during which the parachuting technique [6] was employed. The proximal humeral fracture was approached via deltopectoral approach initially stabilized using a locking plate restoring the normal anatomical alignment of the proximal humerus, followed by addition of a buttress plate to support the calcar (Figure 4). The comminuted olecranon fracture was fixed using multimodal strategies including bridging plates, cerclage wire, K-wires and headless screws (Figure 4). The left sacral non-displaced fracture was managed conservatively. Neurovascular integrity was maintained throughout.

**Figure 4:**
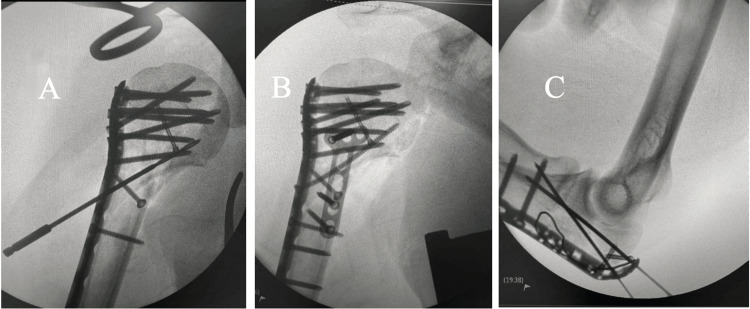
Intraoperative fluoroscopic images showing definitive fixation A: Oblique view of the humeral fixation demonstrating screw placement and construct stability, B: Dual plating of the proximal humerus with restoration of humeral head alignment and medial calcar support, C: Fixation of the comminuted olecranon with bridging plate, cerclage wire, and K-wires

Postoperatively, the patient was managed according to the Enhanced Recovery After Surgery (ERAS) protocol [7], incorporating multimodal analgesia, early mobilization, and coordinated rehabilitation. The immediate postoperative course was uneventful, with no complications or signs of infection. Postoperative radiographs confirmed satisfactory fixation, with dual plating of the proximal humerus (Figure 5) and multimodal fixation of the comminuted olecranon using a combination of plates, cerclage wiring, and K-wires (Figure 6). The patient was discharged independent in activities of daily living. At the six-month follow-up, clinical and functional outcomes were satisfactory: elbow range of motion was maintained between 5° and 140°, and shoulder rehabilitation had been initiated with encouraging recovery. Functional progress was further demonstrated clinically, with images showing an active range of motion of the shoulder and elbow (Figure 7). Psychological evaluation revealed a moderate risk of self-harm; however, the patient remained oriented, cooperative, and compliant with rehabilitation. 

**Figure 5:**
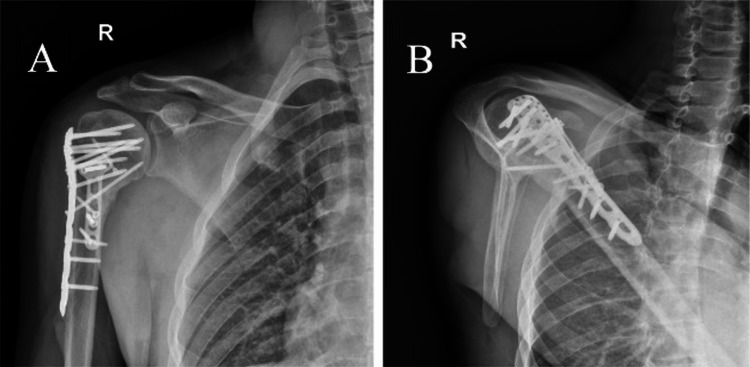
Postoperative radiograph of the right proximal humerus showing dual-plate fixation A: Anteroposterior view of the humerus, B: Lateral view of the humerus

**Figure 6:**
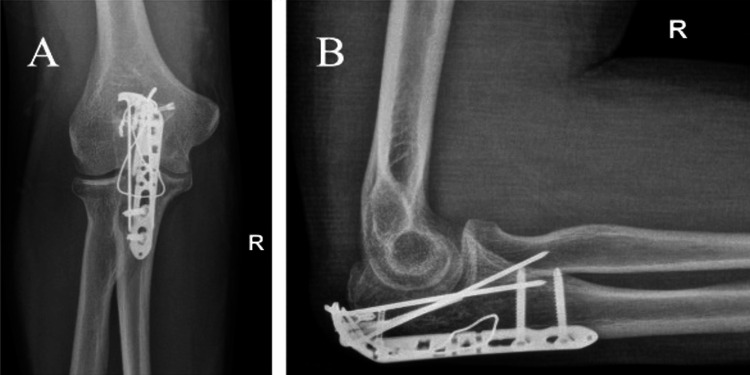
Postoperative radiograph of the right elbow showing multi-modal fixation of the comminuted olecranon fracture A: Anteroposterior view of the olecranon, B: Lateral view of the olecranon

**Figure 7:**
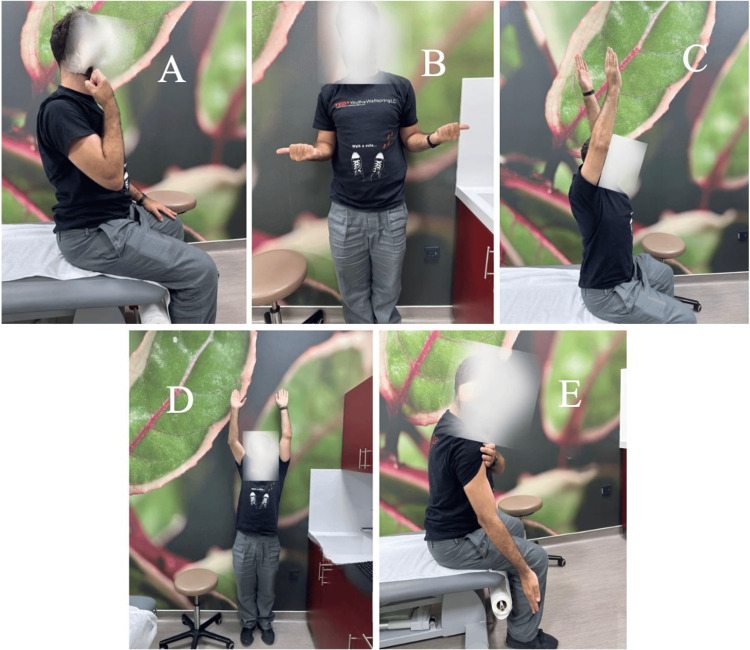
Postoperative range of motion of the right upper limb at the sixth month review A: Elbow flexion, B: Forearm supination, C: Seated overhead elevation, D: Standing overhead elevation, E: Cross-body adduction/shoulder horizontal adduction The patient maintained functional range (elbow range of motion: 5° to 140°).

## Discussion

This case illustrates the challenges of managing complex open proximal humerus and ipsilateral proximal ulna fractures in a young, intoxicated polytrauma patient. On presentation, the patient was hypotensive and tachycardic, emphasizing the critical importance of adhering to ATLS principles to stabilize life-threatening physiology before addressing limb injuries [8]. Once resuscitated, fracture classification guided surgical planning. The proximal humerus injury was a three-part displaced fracture, as defined by Neer’s system [9]. Recognition of displacement and potential compromise of humeral head vascularity, as highlighted by Hertel et al., informed the decision to pursue operative fixation rather than conservative management, given the high risk of avascular necrosis if left untreated [10].

Operative management prioritized stability and anatomical reduction. In this patient, dual plating of the proximal humerus with calcar was performed to enhance medial support and minimize the risk of varus collapse, consistent with biomechanical principles described by Neer [11]. To further optimize fixation, the parachuting technique [6] was employed, allowing multidirectional screw placement to enhance stability and reduce the risk of fixation failure [12]. The proximal ulna, being comminuted, required a multimodal fixation strategy, incorporating plating, cerclage wires, and K-wires, to achieve stable alignment and allow early mobilization, reflecting the complexity of the injury and techniques recommended for multi-fragmentary upper extremity fractures. Careful soft tissue handling and restoration of anatomical alignment helped preserve neurovascular integrity and optimized the conditions for early rehabilitation.

The open nature of the fracture (Gustilo type 2) [5] necessitated timely irrigation and debridement to minimize infection risk. Evidence suggests that prompt debridement, together with early antibiotic administration, is more important than adherence to a strict 'six-hour rule' [13,14]. In this patient, early surgical intervention and staged definitive fixation 48 hours later aligned with these principles and contributed to an uncomplicated postoperative course.

An additional layer of complexity in this case was the patient’s intoxication at presentation, which rendered him temporarily incapable of providing valid consent. Emergency surgical intervention, therefore, required reliance on surrogate decision-making in accordance with established ethical and legal frameworks. By obtaining informed consent from the patient’s sibling and securing formal approval from two senior consultants, the treating team acted in line with the principles of the Mental Capacity Act 2005 [15]. This highlights the importance of clear protocols for safeguarding patient autonomy while enabling timely life- and limb-saving interventions in situations where capacity is impaired.

Early rehabilitation was initiated once stable fixation was confirmed, consistent with recommendations for proximal humeral fracture recovery [16-17]. Incorporation of ERAS principles [7], such as early mobilization, multimodal analgesia, and optimization of perioperative care, further supported functional recovery [18]. The patient achieved meaningful functional improvement in shoulder and elbow mobility, demonstrating the value of coordinated multidisciplinary care even in complex polytrauma cases.

This injury pattern, combining an open three-part proximal humeral fracture with an ipsilateral comminuted olecranon fracture, is exceedingly rare in the existing literature. In a multicenter cohort of 78 proximal ulna fractures, associated ipsilateral injuries were reported in 22% of cases, most commonly involving the proximal radius, with no documented cases of concurrent proximal humerus fractures [19]. Similarly, epidemiological studies of proximal humeral fractures describe associations with clavicular and scapular injuries but not ipsilateral ulna involvement [1]. The absence of this combined pattern in large clinical series suggests it is either exceptionally uncommon or under-recognized in the context of high-energy polytrauma. This case, therefore, adds to the limited literature on complex ipsilateral upper limb injury patterns and highlights the importance of thorough assessment in patients sustaining high-energy trauma, where multiple anatomically distinct fractures may coexist and be easily overlooked.

This case highlights how high-energy polytrauma may present with rare ipsilateral upper limb fracture combinations requiring concurrent prioritization of resuscitation, definitive fixation strategy, and ethical decision-making. It reinforces the importance of adherence to ATLS principles in hemodynamically unstable patients, timely surgical planning in the context of impaired capacity due to intoxication, and the role of stable fixation using dual plating, calcar support, and multimodal techniques. Early, coordinated rehabilitation remains essential in optimizing functional outcomes even in complex upper limb trauma.

## Conclusions

This case demonstrates the successful management of a rare ipsilateral upper limb fracture pattern through timely ATLS-based resuscitation, early debridement, and stable dual-plating fixation with multidisciplinary input. Ethical challenges related to impaired consent due to intoxication were managed using best-interest principles. Early rehabilitation contributed to satisfactory functional recovery with preserved neurovascular integrity.
